# Acute Deterioration in a Ventilated Preterm Infant

**DOI:** 10.1111/jpc.70452

**Published:** 2026-05-27

**Authors:** Nidhi Anand, Atul Malhotra, Pramod Pharande

**Affiliations:** ^1^ Department of Paediatrics Monash University Melbourne Australia; ^2^ Monash Newborn Monash Children's Hospital Melbourne Australia

## 1 Case

A male neonate, second of a dichorionic diamniotic twin pregnancy, was born prematurely at 29^+4^ weeks gestation with hydrops fetalis of unknown aetiology. Bilateral pleural effusions diagnosed at 24 weeks were managed with in utero drains, which subsequently dislodged. At birth, he required intubation and left‐sided chest drain insertion, followed by a right drain at 15 h for persistent effusion. At 18 h of age, he acutely deteriorated with bradycardia and hypotension. Chest X‐ray initially showed persistent left‐sided pneumothorax and intracardiac air necessitating placement of a second left drain. Air was also aspirated from the arterial line. Despite extensive resuscitation, he died. Post‐mortem examination was declined by parents. What is the diagnosis?

## 2 Answer

**FIGURE 1:**
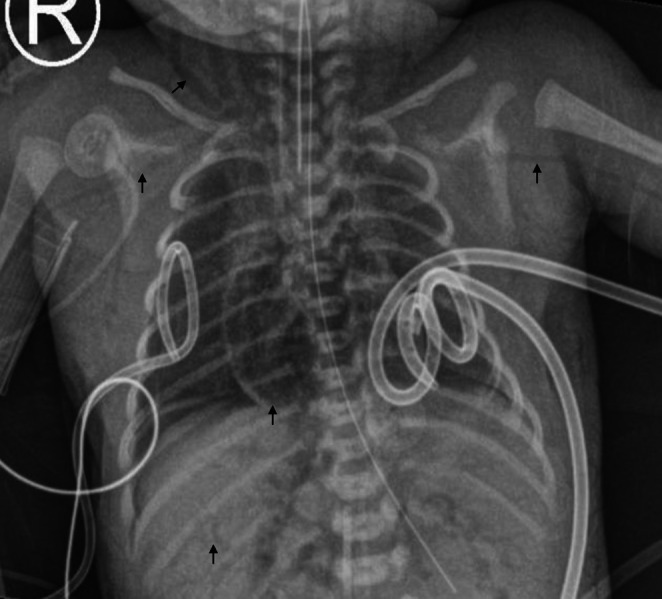
Chest X‐ray showing extensive vascular air embolism involving the heart, subclavian arteries, neck and abdominal vessels (arrows).

Repeat chest X‐ray indicated extensive vascular air embolism involving the heart, subclavian arteries, neck and abdominal vessels (Figure 1).

While rare, vascular air embolism in neonates is frequently fatal [1]. Risk is highest in mechanically ventilated preterm neonates due to barotrauma and microscopic alveolar rupture. Preceding air‐leak syndromes, including pneumothorax and pneumomediastinum, further worsen prognosis [1, 2]. Moreover, preterm newborns requiring prolonged cardiopulmonary resuscitation may be particularly vulnerable owing to the fragility of immature lung tissue [3, 4]. Radiographically, intravascular air may be seen within the heart and can extend through both the arterial and venous systems [1, 5].

Given the high mortality and risk of adverse neurological sequelae, the early recognition and prevention of vascular air embolism in neonates is critical [1]. Acute clinical deterioration of a ventilated preterm neonate should raise a high index of suspicion, warranting urgent imaging. Preventative management includes minimising mechanical ventilation, correcting air leaks promptly, Trendelenburg and left decubitus positioning, and administering 100% oxygen [1, 5].

## Funding

The authors have nothing to report.

## Ethics Statement

The authors declare that the research presented in this report adheres to the ethical principles outlined by the Monash Health Research Ethics Committee. All procedures involving human participants were conducted in accordance with the ethical standards of the institution and the Declaration of Helsinki (1964), as revised in 2013. Parental consent was obtained to share the de‐identified image and clinical details.

## Consent

Parental consent was obtained for sharing this de‐identified report.

## Conflicts of Interest

The authors declare no conflicts of interest.

## Data Availability

The data that support the findings of this study are available on request from the corresponding author. The data are not publicly available due to privacy or ethical restrictions.
